# A Star Recognition Method Based on the Adaptive Ant Colony Algorithm for Star Sensors

**DOI:** 10.3390/s100301955

**Published:** 2010-03-10

**Authors:** Wei Quan, Jiancheng Fang

**Affiliations:** Novel Inertial Instrument and Navigation System Technology Laboratory, School of Instrumentation Science and Optoelectronics Engineering, Beijing University of Aeronautics and Astronautics, Beijing 100191, China; E-Mail: fangjiancheng@buaa.edu.cn

**Keywords:** star sensor, ant colony algorithm, star recognition, guidance-star database

## Abstract

A new star recognition method based on the Adaptive Ant Colony (AAC) algorithm has been developed to increase the star recognition speed and success rate for star sensors. This method draws circles, with the center of each one being a bright star point and the radius being a special angular distance, and uses the parallel processing ability of the AAC algorithm to calculate the angular distance of any pair of star points in the circle. The angular distance of two star points in the circle is solved as the path of the AAC algorithm, and the path optimization feature of the AAC is employed to search for the optimal (shortest) path in the circle. This optimal path is used to recognize the stellar map and enhance the recognition success rate and speed. The experimental results show that when the position error is about 50″, the identification success rate of this method is 98% while the Delaunay identification method is only 94%. The identification time of this method is up to 50 ms.

## 1. Introduction

In order to supply precise attitude for control systems, almost all spacecrafts need to obtain the attitudes. There are several sensors to determine the attitude relative to reference objects. Star sensors are the most effective among them, acquiring the attitude information by star map processing methods and attitude-determining algorithms.

An autonomous star recognition method is one of the core technologies of spacecraft attitude measurements with a star sensor. According to the original star map data obtained by the star sensor, the identification method transforms, transfers or combines the star points, which are included in a star map, and comes up with the characteristic information that reflects this star map as far as possible. Then, the information is compared with the Guidance-star database to complete the identification of the star map. The identification method must be able to achieve the rapid acquisition of spacecraft attitude, as well as rapid attitude reconstruction. Therefore, the speed and success rate of identification are the key factors for judging the performance of identification algorithms.

There are many popular star map recognition methods, which can be divided into three groups: the graph theory-based, the primary star-based and the intelligence-based. For example, the triangular [1,2], the quadrilateral [3] and the Delaunay triangulation [4] fall into the first group. The planar triangles [5], the grid [6], the identification method based on constellation [7], the statistical characteristics [8], the hausdorff distance [9] and the pyramid algorithm [10] fall into the second group. Finally, the star pattern recognition method based on neural network [11] and the genetic algorithm [12] fall into the third group. These algorithms have their respective merits in recognition speed, recognition success rate, the sky coverage rate, the database size, antinoise performance and stability. But in the condition of large Field Of View (FOV), which is no less than 20° × 20° and high-sensitivity, the recognition speed and success rate are the two outstanding problems for star recognition methods.

The ant algorithm (AA) was first proposed by Italian scholar M. Dorigo in 1991. It is an optimization algorithm for simulating the foraging behavior of ant groups [13]. The AA uses parallel self-catalytic mechanism of positive feedback and has strong robustness, good distributed computing capacity and quick optimal (shortest) path searching ability. In recent years, in order to improve the performance of the ant colony algorithm, some improved ant colony algorithms are presented by other scholars [14–20]. Among them, the Adaptive Ant Colony (AAC) algorithm not only has the ability of a global search, but also can effectively restrain the local convergence and prematurity [17,19]. At present, it has been widely used in the vehicle routing problem, cluster analysis, image processing, data mining, track layout optimization and planning. The AAC algorithm is firstly introduced into star identification in this paper and successful results can be achieved.

## 2. The Star Recognition Method Based on AAC Algorithm

To address the problem of many star points caused by the large FOV and the high sensitivity of the star sensor, an AAC algorithm is introduced to the star recognition, which has the parallel processing capabilities and features of fast path optimization. First, the algorithm calculates the average gray value of the set of star points and chooses the star points that meet the average gray value to form the new set of star points. Many circles are drawn that are centered on each star point in this set and the radius is set to a special angular distance, which is less than half of FOV. Then a set including all star points in every circle is composed. The angular distance of every two star points in each star point set is calculated and marked as the path of ants. There is a unique and shortest path that passes through all star points in every star point set. If the AAC parameters can be properly selected and the size of star points is appropriate, using the fast path optimization capability of the AAC algorithm, the shortest (optimal) path can be found rapidly. In the same way, the Guidance-star database can be constructed by AAC algorithm. At last, the star pattern recognition can be quickly finished by matching the shortest path between star-point set and the Guidance-star database, which stores many shortest paths optimized by the AAC algorithm previously and has small, non-redundant capacity. The experimental results show that successful results can be achieved. Namely, this method is fast and robust, and has a high recognition success rate, and only needs a small database, which is better than the star recognition based on Delaunay cutting algorithm and the improved triangle identification.

### 2.1. The Principle of the AAC Algorithm

Scientists have found that although there is no visual sense for each ant, the optimal path would be found by the pheromone, which is released by ants at movement [20]. As social insects, the ants in the colony transfers information to each other through pheromones and cooperate to complete complex tasks. The ant colony has good learning and adapting capability to environmental changes [21,22]. The theory of the specific ant colony algorithm can be described as follows.

Suppose *G* is set as the food sources that will be found by the entire ant colony, *G* = {*g*|1,2,…,*M*}. *S* is the ant set, *N* is the number of ants, *S* = {*s*|1,2,…,*N*}. *g_ij_* denotes the distance from food source *i* to food source *j*, namely the distance of the path (*i*,*j*), where *i*,*j* ∈ *G*. *C_i_*(*t*) denotes the number of ants at time *t* at the food source *i*, $N=\sum_{i=1}^{M}C_{i}(t)$.

*τ_ij_*(*t*) denotes the amount of pheromone on the path (*i*,*j*) at time *t*. The amount of pheromone is equal on every path at initial time, set *τ_ij_*(0) is a constant. The table *u_k_*(*k* = 1,2,…,*N*) is used to store the visited food source. During the movement of the ant *k*, the ant chooses the path according to the transition probability, which is calculated by using the amount of pheromone and heuristic information of ant path, $p_{\mathrm{ij}}^{k}(t)$ denotes the transition probability of the ant from food source *i* to food source *j* at time *t*.

(1) $$p_{\mathrm{ij}}^{k}(t)=\{\begin{array}{ll} \frac{{\left[\tau_{\mathrm{ij}}(t)\right]}^{\alpha }\;{\left[\eta_{\mathrm{ij}}(t)\right]}^{\beta }}{\sum_{s\in d_{k}}{\left[\tau_{\mathrm{is}}\;(t)\right]}^{\alpha }\;{\left[\eta_{\mathrm{is}}\;(t)\right]}^{\beta }}, & j\in d_{k}\\ 0, & j\notin d_{k} \end{array}$$

Where *d_k_* = {*G* − *u_k_*} indicates the food sources for the ant *k* to choose. *α* and *β* are two constants that respectively define the influence of heuristic component and the trail information on decision of ants [15]. *η_ij_*(*t*) is the heuristic function; *η_ij_*(*t*) = 1/*g_ij_* indicates the expected time of the ant from food source *i* to food source *j*. With the movement of the ant colony, the pheromone released previously will gradually desalt. After time *T* has passed, all ants will complete one circulation, and the amount of pheromone will be updated by the following formula on the path (*i*,*j*) at the time *t* + *T*:

(2) $$\tau_{\mathrm{ij}}(t+T)=(1-\rho )\tau_{\mathrm{ij}}(t)+\Delta \tau_{\mathrm{ij}}(t),\Delta \tau_{\mathrm{ij}}(t)=\sum_{k=1}^{N}\Delta \tau_{\mathrm{ij}}^{k}(t)$$

Where *ρ* is the information volatile factor. Δ*τ_ij_*(*t*) is the increment of pheromone on the path (*i*,*j*) during this circulation, Δ*τ_ij_*(0) = 0 at initial time. $\Delta \tau_{\mathrm{ij}}^{k}(t)$ is the number of pheromone which is released by the ant *k* on the path (*i*,*j*) in this circulation [17].

(3) $$\Delta \tau_{\mathrm{ij}}^{k}(t)=\{\begin{array}{ccc} \frac{\lambda_{k}Q}{L_{k}}, & & T\\ 0, & \mathrm{not} & T \end{array},\lambda_{k}=\{\begin{array}{ll} (L_{\mathrm{avg}}-L_{k})/(L_{\mathrm{avg}}-L_{\mathrm{opt}}), & L_{k}<L_{\mathrm{avg}}\\ 0, & L_{k}\geq L_{\mathrm{avg}} \end{array},L_{\mathrm{avg}}=\frac{1}{N}\sum_{k=1}^{N}L_{k},L_{\mathrm{opt}}=\underset{k=1,2,\cdots ,N}{\text{min}}(L_{k})$$

Where *Q* is the extent of pheromone, the default is a constant, which affects the rapidity of convergence of algorithm at the extent. *L_k_* is the total length that the ant *k* participates in this circulation. *T* denotes the ant *k* passes the path (*i*,*j*) in this circulation.

### 2.2. The Construction of the Guidance-Star Database Based on the AAC Algorithm

The Guidance-star database is a unique basis for star map identification of a star sensor. The speed of identification depends to some extent on the capability and searching strategy of the Guidance-star database in addition to the processor. The Guidance-star database stores the data of star point recognition and star declination, right ascension, star magnitude, *etc*. It is commonly loaded in the memory of a star sensor. The star declination, right ascension, and star magnitude are generally obtained from the basic star catalog, which is downloadable. The data of star point recognition is closely related to the star recognition algorithm.

**Figure 1.**
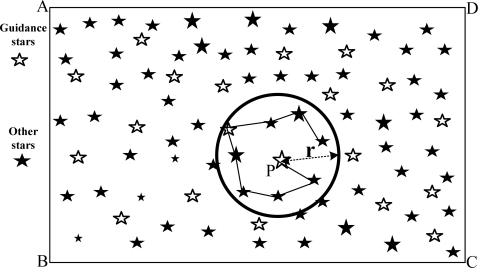
Schematic plan of the optimal path from path optimization using the AAC.

Different star recognition methods can construct variant Guidance-star databases, especially the part that is used for star point recognition, determining the storage capacity of the Guidance-star database. Aiming at the star recognition method based on the adaptive ant colony algorithm, the construction of the Guidance-star database is as follows:

1. Select guidance stars. In order to guarantee existing guidance stars in random FOV, the screening of all stars is carried out. According to the spectral response curve of sensitive chip, optical lens and sensitivity of the star sensor, some stars will be selected from the basic star catalog to cover the overall celestial sphere as far as possible [23].
2. For any guidance star *i*, a circle is drawn that is centered on the guidance star *i*, with radius *r*, which is used to guarantee at least three stars in the circle for the Guidance-star database construction based on AAC algorithm. If the guidance star *j* meets the formula (4), it will be put into the star point set.
  (4) $$r>\theta =\text{arccos}(\frac{\bar{i}•\bar{j}}{\left|\bar{i}\right|\left|\bar{j}\right|})$$
  Where, θ is the angular distance of star i̅ and j̅ star in the celestial coordinate system, arccos() means anti-cosine function, i̅ · j̅ means dot product between i̅ and j̅.
3. For the stars in each star point set, the angular distance of any pair of star points in the star sets is calculated and the star point closest to the center of the star point set as the starting point is taken. Then the optimal (shortest) path of this star point set is retrieved with the AAC. Suppose the star point *P* is the starting point, then the path in Figure 1 shows the optimal path.
4. At last, the angular distance values of the path optimization for each star point set are stored in an ascending-order database as a record. At the same time, the coordinate information of the stars is stored as well. Finally, the Guidance-star database is constructed. Under the condition of guaranteeing a successful rate of identification, and referring to the triangle identification algorithm (which only uses three angular distance paths), and also considering the memory load of the Guidance-star database, the only two angular distance paths and three star points’ sequence are involved. The Guidance-star database will be constructed by this method, without redundant data and with a smaller capacity than the triangle identification method, which needs some redundant triangles. Because this database stores angular distances with ascending order, the quick look-up algorithm and the binary search algorithm can be used to further enhance the identification speed.

The part of information stored in the Guidance-star database constructed by this method is shown in Table 1.

### 2.3. The Star Recognition Based on the AAC Algorithm

The AAC algorithm has the ability of global searching and effectively restrains the local convergence and parallel processing ability, *etc.* If the star recognition method based on the AAC algorithm meets the following precondition, it can obtain good effect.

(1) The precondition of recognition algorithm.

This recognition algorithm is suitable to the star recognition for the star sensor with a large FOV, which is no less than 20° × 20° and is highly sensitivity, with no less than 6.90 magnitude. The precondition of recognition algorithm is that the highest magnitude of star detected by the star sensor should be no more than the highest instrumental magnitude of the star in the Guidance-star database.

(2) Using the adaptive ant colony algorithm to identify a star map quickly.

The most important reason for applying this recognition algorithm based on the adaptive ant colony algorithm to recognize a star map is that each star map has a feature—except for the uncertainty factors such as background flashes from satellites and space junks and cosmic ray hits, *etc.*—every star map should be a subset of the all-sky star map.

**Figure 2.**
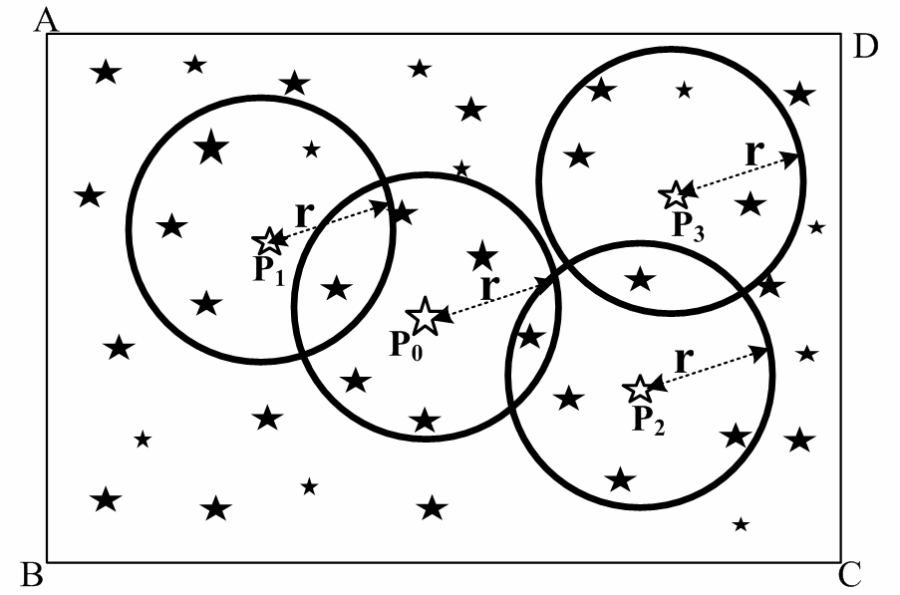
The results after step 1) and step 2) of the adaptive ant colony algorithm used to identify a star map.

**Figure 3.**
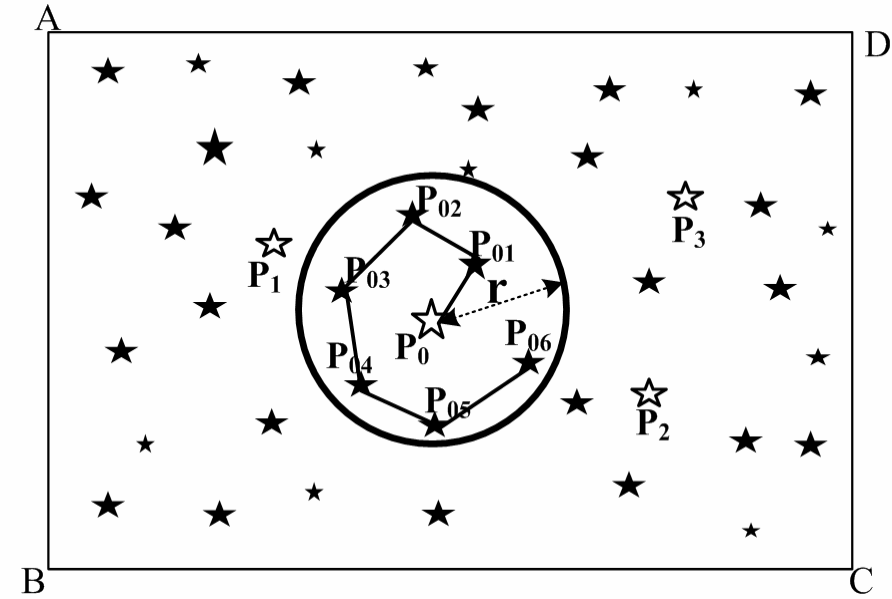
The Optimal route map of light points in circle by the AAC.

There is a star map *IMG*, which includes *Q* light points. The point set *S* is composed of the *Q* light points, *S* = {*s_1_*, *s_2_*, ..., *s_Q_*}. Using the adaptive ant colony algorithm to identify the star map *IMG*, the steps are as follows:

1. The average gray value *D_avg_* of point set *S* is calculated. Suppose the centroid position of element *s_i_* is (*x_i_*,*y_i_*), whose pixel value is *Vx_i_y_i_*, the intensity *Ds_i_* of element *s_i_* is calculated by the following formula. Here, a circle average is taken for the star intensity of which the radius is three pixels.
  $$D_{s_{i}}=\sum_{p=x_{i}-2}^{x_{i}+2}\sum_{q=y_{i}-2}^{y_{i}+2}V_{\mathrm{pq}}+V_{x_{i}-3,y_{i}}+V_{x_{i},y_{i}-3}+V_{x_{i}+3,y_{i}}+V_{x_{i},y_{i}+3}$$
  So, the *D_avg_* is
  $$D_{\mathrm{avg}}=\frac{1}{Q}\cdot \sum_{j=1}^{Q}D_{s_{j}}$$
  Selecting the light points whose value *D* and angular distance *ϕ* between itself and the center point of the star map meet the following formula (5) to form the light point set *T*, *T* = {*t_1_*, *t_2_*, ..., *t_R_*}, *R* is the size of set *T*.
  (5) $$\left(D>D_{\mathrm{avg}})\cup (\phi +r\leq \frac{\mathrm{Fov}}{2}\right)$$
  Here, *Fov* is the FOV of the star sensor.
2. Centering at every light point of the point set *T*, a circle is drawn with a radius equal to angular distance *r*, centered on this light point. Figure 2 is the results of step (1) and step (2) for a star map. There are four light points (*P*_0_, *P*_1_, *P*_2_ and *P*_3_), which meet the formula (5).
3. Select a light point (*P*_0_), which is nearest to the center of the star map from *P*_0_, *P*_1_, *P*_2_ and *P*_3_. There is a circle drawn with the center as light point *P*_0_ and the radius equal to special angular distance *r*. The light points that belong to the circle form the light point set *M*, and the angular distance of every two light points is found in *M*. Then the path optimization of *M* is accomplished by the AAC. Figure 3 is the result of optimization.
4. The angular distance values of *P*_0_*P*_01_ and *P*_01_*P*_02_ are stored and compared respectively with angular distance *d*_1_ and angular distance *d*_2_ of the Guidance-star database. If the inequality (6) is true, the result of identification is successful.
  (6) $$\sqrt{{\left(P_{0}P_{01}-d_{1}\right)}^{2}+{\left(P_{01}P_{02}-d_{2}\right)}^{2}}<\theta_{\text{th}}$$
  Here, *θ*_th_ is the minimal threshold of identification, the default value is the mean square deviation of system error.
  Otherwise, the light point *P*_1_ is selected and a circle is drawn with the center as the light point *P*_1_ and the radius being the angular distance *r*. Then step (3) and step (4) are repeated. If the light points *P*_2_ and *P*_3_ do not meet the inequality (6) until no other light points are selected, the result of identification is unsuccessful.

## 3. Hybrid Simulation Result and Analysis

In this paper, the FOV is set to 20° × 20°, and the resolution is set to 1024×1024 pixels. For the purpose of validation of the presented method, the fundamental star catalog is composed of 14,581 stars, which are selected from the Tycho2 star catalog. The highest magnitude detected by the star sensor is 6.95. The range of star position error is set to less than 120″ according to the precision of star centroid extraction.

In regard to the problem of how to choose the ACC parameters, according to reference [22], the maximal cycle index is commonly set to 1,0000/*N* (the number of ants), *α* and *β* belong to [0, 5] (default *α* = *β* = 1), the *Q* can be set in the range from 0 to 7,000, and the volatile coefficient of pheromone *ρ* is always in the range of [0.1, 0.99] (which relates to the ability of global optimality and the convergence speed; the bigger ρ is, the faster the speed, the easier the local optimality, and the more difficult the global optimality). According to the FOV and the highest magnitude, we know that there are about 50 stars in every star map, the number of ants *N* is set to 50 and the initial maximal cycle index is set to 200. Combining the experimented data, *α* and *β* are respectively set to 1; the intensity of pheromone *Q* in every path is set to 2000; the *ρ* is set to 0.2; and the angular distance *r* is set to 1/12 * FOV to guarantee the global optimality and uniqueness of results.

**Figure 4.**
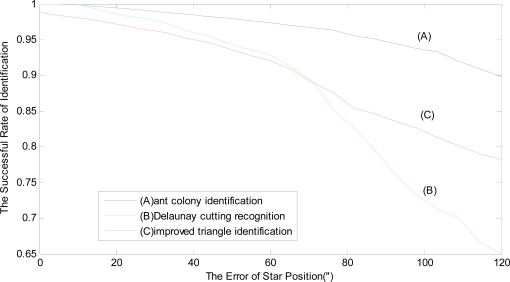
Relationship between star position error and success rate of identification.

The hybrid simulation is carried out for this identification method. Considering the hybrid simulation time, 10 stochastic optic axes are produced by the stochastic toss of Monte Carlo method. For the every optic axis, there is a standard star map. And for every standard star map (total of 10 star maps), the star position error, which is from 0″ to 120″, is added in the star map. There are 120 error points of star position for hybrid simulation. For every error point, 100 trials of the identification method are carried out and the mean value of the successful rate of identification is calculated as this error point’s successful rate of identification. Finally, the hybrid simulation results of ten star maps are averaged, according to the every error point. The average value of the successful rate is shown in Figure 4. Figure 4 (A) shows the relationship between position error and success rate of the proposed identification. During the course of hybrid simulation, compared with the star recognition based on Delaunay cutting algorithm [4] (B) and the improved triangle identification [1] (C), the results of hybrid simulation are as follows (the Delaunay identification data and improved triangle data originate from reference [4]).

As seen in Figure 4, when the position error is small (less than 12″), the identification success rate of this method is about 99.5% and equivalent to the Delaunay identification method. Because the Delaunay identification adopts the angular distance information of three vertices for cutting triangle to identify star map, when the position error gradually becomes larger, the accuracy of the Delaunay identification is quickly falling. However, the identification success rate of the star recognition based on the adaptive ant colony algorithm is not sensitive to the position error and still keeps a high success rate of recognition. When the position error is about 50″, the identification success rate of this method is about 98%, while the other two identification methods are only about 94%. As a whole, it can be seen from Figure 4 that the recognition success rate of this method is higher than the Delaunay identification method and the improved triangle identification method.

In regard to the aspect of recognition speed, the identification time is mainly relative to the performance of the AAC algorithm, the capacity of the Guidance-star database, the star position error, and the number of stars per image. A main factor is that the AAC algorithm has a good parallel processing ability to improve the recognition speed. The Guidance-star database adopts the storage mode of database in this method, and the angular distance optimization results of the basic star table by using the adaptive ant colony algorithm are stored in the ascending-order database, which has small data redundancy. Here the binary search algorithm is used. Thus, the star position error is one of the important factors affecting the identification time of the AAC identification method.

**Figure 5.**
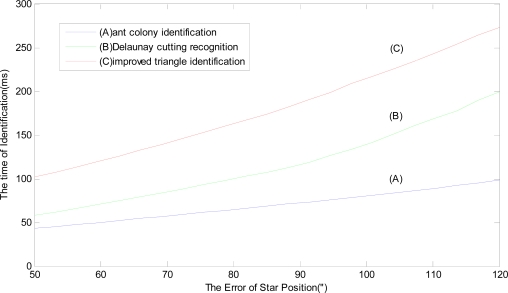
Relationship graph of position error and the time of identification.

Because the star position error is less than 50″, the time of identification changes very slightly for the three identification methods. For every standard star map (total 10 star maps), the star position error, which is from 50″ to 120″, is added in the star map. There are 70 error points of star position for hybrid simulation using two AT91RM9200 180 MHz digital processors, which are linked to each other with Linear Array Structure. Similarly, for every error point of star position, 100 trials are still carried out and the mean value of the execution time of identification is calculated as this error point’s execution time. Finally, we still average the hybrid simulation results of 10 star maps according to every error point. The average value of the execution time is shown in Figure 5, which shows the relationship of position error and the time of identification. The results of the star recognition based on the Delaunay cutting algorithm and the improved triangle identification are shown respectively in Figure 5B and Figure 5C.

As seen in Figure 5, when the error of star position is 50″ and under the same condition for the three star identification methods, the identification time of (A) is less than 50 ms, because of the parallel processing ability The identification time of (B) is more than 50 ms while (C) is more than 100 ms because of the processing ability and the large capacity of the Guidance-star database. With the augmentation of the error of star position, the identification time gradually becomes longer. The growth rate of the identification time of (A) is the slowest among the three methods. When the error of star position is 120″, (A) is about 100 ms, because it is not sensitive to the position error, while (B) and (C) are, respectively, about 200 ms and 280 ms.

## 4. Conclusions

A new star recognition method is presented in this paper. This method employs an AAC algorithm to search optimal angular distance of star points set, completes the angular distance optimization of star points set, and achieves quick identification of a star map. This identification method calculates the angular distance of any pair of star points by the parallel processing ability of the AAC algorithm to improve the speed of identification. The angular distance of two star points is solved as the path of the AAC algorithm. The optimal path is found by the AAC path optimization characteristics and is used to recognize the stellar map and enhance the recognition success rate. The presented method can solve the problems of star recognition speed and reduced success rate due to a large FOV and high sensitivity of a star sensor, and overcomes the drawback of redundant matches because of small feature dimensionality for the triangle identification and deficiency caused by the Delaunay identification sensitive to the position error. Hybrid simulation results show that when the measured position error is about 50″, the identification success rate of this method still keeps 98%, while the Delaunay identification method is about 94%, and the identification time of this method is merely 50 ms. So the method has a high recognition success rate, and is fast and robust. It only needs a small database and has fast search speed.

## Figures and Tables

**Table 1.** Part of data stored in the Guidance-star database.

Serial number	Angular distance 1	Angular distance 2	Star point 1	Star point 2	Star point 3
	Degree/°		(right ascension/°, declination/°)		
2558	1.9001	1.9385	(185.55,24.774)	(187.23,25.913)	(185.07,26.002)
2559	1.9009	2.0574	(185.08,26.620)	(187.16,26.227)	(185.55,24.774)
2560	1.9013	2.1628	(187.19,25.899)	(185.07,26.002)	(184.87,28.157)
